# Automatic Classification of Online Doctor Reviews: Evaluation of Text Classifier Algorithms

**DOI:** 10.2196/11141

**Published:** 2018-11-12

**Authors:** Ryan Rivas, Niloofar Montazeri, Nhat XT Le, Vagelis Hristidis

**Affiliations:** 1 Department of Computer Science and Engineering University of California, Riverside Riverside, CA United States

**Keywords:** patient satisfaction, patient reported outcome measures, quality indicators, health care, supervised machine learning

## Abstract

**Background:**

An increasing number of doctor reviews are being generated by patients on the internet. These reviews address a diverse set of topics (features), including wait time, office staff, doctor’s skills, and bedside manners. Most previous work on automatic analysis of Web-based customer reviews assumes that (1) product features are described unambiguously by a small number of keywords, for example, *battery* for phones and (2) the opinion for each feature has a positive or negative sentiment. However, in the domain of doctor reviews, this setting is too restrictive: a feature such as *visit duration* for doctor reviews may be expressed in many ways and does not necessarily have a positive or negative sentiment.

**Objective:**

This study aimed to adapt existing and propose novel text classification methods on the domain of doctor reviews. These methods are evaluated on their accuracy to classify a diverse set of doctor review features.

**Methods:**

We first manually examined a large number of reviews to extract a set of features that are frequently mentioned in the reviews. Then we proposed a new algorithm that goes beyond bag-of-words or deep learning classification techniques by leveraging natural language processing (NLP) tools. Specifically, our algorithm automatically extracts dependency tree patterns and uses them to classify review sentences.

**Results:**

We evaluated several state-of-the-art text classification algorithms as well as our dependency tree–based classifier algorithm on a real-world doctor review dataset. We showed that methods using deep learning or NLP techniques tend to outperform traditional bag-of-words methods. In our experiments, the 2 best methods used NLP techniques; on average, our proposed classifier performed 2.19% better than an existing NLP-based method, but many of its predictions of specific opinions were incorrect.

**Conclusions:**

We conclude that it is feasible to classify doctor reviews. Automatically classifying these reviews would allow patients to easily search for doctors based on their personal preference criteria.

## Introduction

### Background

The problem of automatic reviews analysis and classification has attracted much attention because of its importance in ecommerce applications [1-3]. Recently, there has been an increase in the number of sites where users rate doctors. Several works have analyzed the content and scores of such reviews, mostly by examining a subset of them through qualitative and quantitative analysis [4-9] or by applying text-mining techniques to characterize trends [10-12]. However, not much work has studied how to automatically classify doctor reviews.

In this study, our objective was to automatically summarize the content of a textual doctor review by extracting the features it mentions and the opinion of the reviewer for each of these features; for example, to estimate if the reviewer believes that the wait time or the visit time is long or if the doctor is in favor of complementary medicine methods. We explore the feasibility of reaching this objective by defining a broader definition of the review classification problem that addresses challenges in the domain of doctor reviews and examining the performance of several machine learning algorithms in classifying doctor review sentences.

Previous work on customer review analysis focused on automated extraction of features and the polarity (also referred as opinion or sentiment) of statements about those features [2,13,14]. Specifically, these works tackle the problem in 2 steps: first they extract the features using rules, and then, for each feature, they estimate the polarity using hand-crafted rules or supervised machine learning methods. This works well if (1) the features are *basic*, such as the battery of a phone, which are generally described by a single keyword, for example, *the battery of the camera is poor*, and (2) the opinion is objectively positive or negative but does not support more subjective features like visit time, where for some patients it is positive to be longer, and for some, it is negative. In other words, statements about features in product reviews tend to be more straightforward and unambiguously positive or negative, whereas reviews on service, such as doctor reviews, are often less so, as there may be many ways to express an opinion on some aspect of the service.

In our study, the features may be more complex, for example, the *visit time* feature can be expressed by different phrases such as “spends time with me,” “takes his time,” “not rushed,” and so on. As another example, “appointment scheduling” can be expressed in many different ways, for example, “I was able to schedule a visit within days” or “The earliest appointment I could make is in a month.” Other complex classes include *staff* or *medical skills*.

Furthermore, in our study, what is positive for one user may be negative for another. For example, consider the sentence “Dr. Chan is very fast so there is practically no wait time and you are in and out within 20 minutes.” The sentiment in this sentence is positive, but a short visit implied by *in and out within 20 minutes* may be negative for some patients. Instead, what we want to measure is long visit time versus short visit time. This is different from work on detecting transition of sentiment [15] because it is not enough to detect the *true* sentiment, but we must also associate it with a class (long visit time vs short visit time).

To address this variation of the review classification problem, we created a labeled dataset consisting of 5885 sentences from 1017 Web-based doctor reviews. We identified several classes of doctor review opinions and labeled each sentence according to the presence and polarity of these opinion classes. Note that our definition of polarity is broader than in previous work as it is not strictly positive and negative but rather takes the subjectivity of patient opinions into account (eg, complementary medicine is considered good by some and bad by others).

We adapt existing and propose new classifiers to classify doctor reviews. In particular, we consider 3 diverse types of classifiers:

1. Bag-of-words classifiers such as Support Vector Machine (SVM) [16,17] and Random Forests [18] that leverage the statistical properties of the review text, such as the frequency of each word.
2. Deep learning methods such as Convolutional Neural Network (CNN) [19], which also consider the proximity of the words.
3. Natural Language Processing (NLP)–based classifiers, which leverage the dependency tree of a review sentence [20]. Specifically, we consider an existing NLP-based classifier [21] and propose a new one, the Dependency Tree-Based Classifier (DTC).

DTC generates the dependency tree for each sentence in a review and applies a set of rules to extract dependency tree–matching patterns. These patterns are then ranked by their accuracy on the training set. Finally, the sentences of a new review are classified based on the highest-ranking matching pattern. This is in contrast to the work by Matsumoto et al [21], which treats dependency tree patterns as features in an SVM classifier.

The results of our study show that classifying doctor reviews to identify patient opinions is feasible. The results also show that DTC generally outperforms all other implemented text classification techniques.

Here is a summary of our contributions:

1. We propose a broader definition for the review classification problem in the domain of doctor reviews, where the features can be complex entities and the polarity is not strictly positive or negative.
2. We evaluated a diverse set of 5 state-of-the-art classification techniques on a labeled dataset of doctor reviews containing a set of commonly used and useful features.
3. We propose a novel decision tree–based classifier and show that it outperforms the other methods; we have published the code on the Web [22].

### Literature Review

In this section, we review research in fields related to this study, which we organize into 5 categories:

- Quantitative and qualitative analysis of doctor review ratings and content
- The application of text mining techniques to describe trends in doctor reviews
- Feature and polarity extraction in customer reviews
- Application of dependency tree patterns to sentiment analysis
- Recent work in text classification

#### Doctor Review Analysis

Several previous works have analyzed Web-based doctor reviews. Gao et al described trends in doctor reviews over time to identify which characteristics influence Web-based ratings [4]. They found that obstetricians or gynecologists and long-time graduates were more likely to be reviewed than other physicians, recent graduates, board-certified physicians, highly rated medical school graduates, and doctors without malpractice claims received higher ratings, and reviews were generally positive. Segal et al compared doctor review statistics with surgeon volume [5]. They found that high-volume surgeons could be differentiated from low-volume surgeons by analyzing the number of numerical ratings, the number of text reviews, the proportion of positive reviews, and the proportion of critical reviews. López et al performed a qualitative content analysis of doctor reviews [6]. They found that most reviews were positive and identified 3 overarching domains in the reviews they analyzed: interpersonal manner, technical competence, and system issues. Hao analyzed Good Doctor Online, an online health community in China, and found that gynecology-obstetrics-pediatrics doctors were the most likely to be reviewed, internal medicine doctors were less likely to be reviewed, and most reviews were positive [7]. Smith and Lipoff conducted a qualitative analysis of dermatology practice reviews from Yelp and ZocDoc [8]. They found that both the average review scores and the proportion of reviews with 5 out of 5 stars from ZocDoc were higher than those from Yelp. They also found that high-scoring reviews and low-scoring reviews had similar content (eg, physician competency, staff temperament, and scheduling) but opposite valence. Daskivich et al analyzed health care provider ratings across several specialties and found that allied health providers (eg, providers who are neither doctors nor nurses) had higher patient satisfaction scores than physicians, but these scores were also the most skewed [9]. They also concluded that specialty-specific percentile ranks might be necessary for meaningful interpretation of provider ratings by consumers.

#### Text Mining of Doctor Reviews

Other previous papers have employed text-mining techniques to characterize trends in doctor reviews. Wallace et al designed a probabilistic generative model to capture latent sentiment across aspects of care [10]. They showed that including their model’s output in regression models improves correlations with state-level quality measures. Hao and Zhang used topic modeling to extract common topics among 4 specialties in doctor reviews collected from Good Doctor Online [11]. They identified 4 popular topics across the 4 specialties: the experience of finding doctors, technical skills or bedside manner, patient appreciation, and description of symptoms. Similarly, Hao et al used topic modeling to compare reviews between Good Doctor Online and the US doctor review website RateMDs [12]. Although they found similar topics between the 2 sites, they also found differences that reflect differences between the 2 countries’ health care systems. These works differ from ours in that they use text-mining techniques to analyze doctor reviews in aggregate, while our goal is to identify specific topics in individual reviews.

#### Customer Review Feature and Polarity Extraction

As discussed earlier in the Introduction, these works operate on a more limited problem setting where the features are usually expressed by a single keyword, and the sentiment is strictly positive or negative. Hu and Liu extracted opinions of features in customer reviews with a 4-step algorithm [2]. This algorithm consists of applying association rule mining to identify features, pruning uninteresting and redundant features, identifying infrequent features, and finally determining semantic orientation of each opinion sentence. Popescu and Etzioni created an unsupervised system for feature and opinion extraction from product reviews [3]. After finding an explicit feature in a sentence, they applied manually crafted extraction rules to the sentence and extracted the heads of potential opinion phrases. This method only works when features are explicit.

#### Sentiment Analysis With Dependency Trees

There are number of existing works that use dependency trees or patterns for sentiment analysis. A key difference is that our method does not always capture sentiment but the various class labels (eg, short or long) for each class (eg, visit time). Hence, we cannot rely on external sentiment training data or on hard-coded sentiment rules, but we must use our own training data.

Agarwal et al used several hand-crafted rules to extract dependency tree patterns from sentences [23]. They combined this information with the semantic information present in the Massachusetts Institute of Technology Media Lab ConceptNet ontology and employed the extracted concepts to train a machine learning model to learn concept patterns in the text, which were then used to classify documents into positive and negative categories. An important difference from our method is that their dependency patterns generally consist of only 2 words in certain direct relations, while our patterns can contain several more in both direct and indirect relations.

Wawer induced dependency patterns by using target-sentiment (T-S) pairs and recording the dependency paths between T and S words in the dependency tree of sentences in their corpus [24]. These patterns were supplemented with conditional random fields to identify targets of opinion words. In contrast to our patterns, which can represent a subtree of 2 or more words, the patterns in this work are generated from the shortest path between the T and S words.

Matsumoto et al’s work [21] is the closest work to our proposed method, which we experimentally compare in the Results section. They extract frequent word subsequences and dependency subtrees from the training data and use them as features in an SVM sentiment classifier. Their patterns involve frequent words and only include direct relations, whereas our patterns involve high-information gain words and consider indirect relations. Pak and Paroubek follow a similar strategy of extracting dependency tree patterns based on predefined rules and using them as features for an SVM classifier [25]. Matsumoto et al perform better on the common datasets they considered.

#### Text Classification

Machine learning algorithms are commonly used for text classification. Kennedy et al used a random forest classifier to identify harassment in posts from Twitter, Reddit, and The Guardian [26]. Posts were represented through several features such as term frequency-inverse document frequency (TF-IDF) of unigrams, bigrams, and short character sequences; URL and hashtag token counts; source (whether the post was from Twitter); and sentiment polarity. Gambäck and Sikdar used a CNN to classify hate speech in Twitter posts [27]. The CNN model was tested with multiple feature embeddings, including random values and word vectors generated with Word2Vec [28]. Lix et al used an SVM classifier to determine patient’s alcohol use using text in electronic medical records [29]. Unigrams and bigrams in these records were represented using a bag-of-words model.

### Problem Definition

Given a text dataset with a set of classes *c*_*1*_, *c*_*2*_, …, *c*_*m*_ that represent features previously identified by a domain expert, each class *c*_*i*_ can take 3 values (polarity):

- *c*_*i*_^*0*^:Neutral. The sentence is not relevant to the class.
- *c*_*i*_^*x*^, *c*_*i*_^*y*^:Yes or no. Note that to avoid confusion, we do not say positive or negative, as for some classes such as *visit time* in doctor reviews, some patients prefer when their visit time is long and some prefer short. In this example, “Yes” could arbitrarily be mapped to *long* and “No” to *short*.

As another example, class *c*_*8*_ from the doctor review dataset is *wait time* or the time spent waiting to see a doctor. It has 3 possible values: *c*_*8*_^*x*^, *c*_*8*_^*y*^, or *c*_*8*_^*0*^. A sentence with class label *c*_*8*_^*x*^ expresses the opinion that the time spent waiting to see the doctor is short. Examples of *c*_*8*_^*x*^ include “I got right in to see Dr. Watkins,” “I’ve never waited more than five minutes to see him,” and “Wait times are very short once you arrive for an appointment.” A sentence with class label *c*_*8*_^*y*^ expresses the opinion that the time spent waiting to see the doctor is long. Examples of *c*_*8*_^*y*^ include “There is always over an hour wait even with an appointment,” “My biggest beef is with the wait time,” and “The wait time was terrible.” A sentence with class label *c*_*8*_^*0*^ makes no mention of wait time. Such sentences may have *c*_*i*_^*x*^ or *c*_*i*_^*y*^ labels from other classes, for example, “This doctor lacks affect and a caring bedside manner” and “His staff, especially his nurse Lucy, go far above what their job requires,” or they may instead not be relevant to any class, such as “Dr. Kochar had been my primary care physician for seven years” and “I’ll call to reschedule everything.” A sentence may take labels from more than one class.

In this study, given a training set *T* of review sentences with class labels from classes *c*_*1*_, *c*_*2*_, …, *c*_*m*_, we build a classifier for each class *c*_*i*_ to classify new sentences to one of the possible values of *c*_*i*_. Specifically, we build *m* training sets *T*_*i*_ corresponding to each class. Each sentence in *T*_*i*_ is assigned a class label *c*_*i*_^*x*^, *c*_*i*_^*y*^, or *c*_*i*_^*0*^.

## Methods

### Doctor Reviews Dataset

We crawled Vitals [30], a popular doctor review website, to collect 1,749,870 reviews. Each author read approximately 200 reviews and constructed a list of features. Afterward, through discussions, we merged these lists into a single list of 13 features, which we represent by classes as described in the problem definition (Table 1).

**Figure 1:**
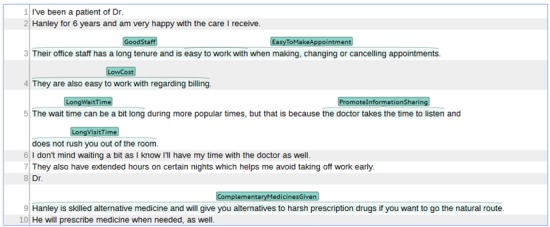
Screenshot of WebAnno’s annotation interface with an annotated review.

To further filter these classes, we selected 600 random reviews to label. We labeled these reviews using WebAnno, a Web-based annotation tool [31] (Figure 1). Specifically, each sentence was tagged (labeled) with 0 or more classes from Table 1 by 2 of the authors. The union of these labels was used as the set of ground-truth class labels of each sentence; that is, if at least one of the labelers labeled a sentence as *c*_*i*_^*x*^, that sentence is labeled *c*_*i*_^*x*^ in our dataset.

We found that some of these classes were underrepresented. For each underrepresented class, we used relevant keywords to find and label more reviews from the collected set of reviews, for example, *wait* for wait time and *listen* for information sharing, which resulted in a total of *1017 reviews* (417 in addition to the original 600). These 1017 reviews are our labeled dataset used in our experiments.

**Table 1:** Description of initial opinion classes. For each class, a sentence that does not mention the class is labeled *c*_*i*_.

Class	*c_i_^x^*	*c_i_^y^*
Appointment scheduling	Easy to schedule an appointment	Hard to schedule an appointment
Bedside manner	Friendly and caring	Rude and uncaring
Complementary medicine	Promotes complementary medicine	No promotion of complementary medicine
Cost	Inexpensive and billing is simple	Expensive and billing problems
Information sharing	Answers questions and good explanations	Does not answer questions and poor explanations
Joint decision making	Treatment plan accounts for patient opinions	Treatment plan made without patient input
Medical skills	Effective treatment and correct diagnoses	Ineffective treatment and misdiagnoses conditions
Psychological support	Addresses stress and anxiety	Does not address stress and anxiety
Self-management	Encourages active management of care	Does not encourage self-management of care
Staff	Staff is friendly and helpful	Staff is rude and unhelpful
Technology	Uses email, Web-based appointments, and electronic health records	Does not use email and Web-based appointments
Visit time	Spends substantial time with patients	Spends very little time with patients
Wait time	Short time spent waiting to see the doctor	Long time spent waiting to see the doctor

**Table 2:** Frequency of each class label in the doctor review dataset.

Class	Frequency of *c_i_^x^*	Frequency of *c_i_^y^*	Frequency of *c_i_^0^*
*c*_*1*_: appointment scheduling	51	84	5750
*c*_*2*_: bedside manner	569	341	4975
*c*_*3*_: cost	25	261	5599
*c*_*4*_: information sharing	316	136	5433
*c*_*5*_: medical skills	481	232	5172
*c*_*6*_: staff	262	368	5255
*c*_*7*_: visit time	143	79	5663
*c*_*8*_: wait time	48	199	5638

Following this, we found that some classes such as complementary medicine and joint decision making were still underrepresented, which we define as having less than 2% of the dataset’s sentences labeled *c*_*i*_^*x*^ or *c*_*i*_^*y*^, so we omitted them from the dataset. The final dataset consists of 5885 sentences and 8 opinion classes. These classes and the frequency of each of their labels are shown in Table 2.

### Background on Dependency Trees

In this section, we describe dependency trees and the semgrex tool that we used for defining matching patterns. Dependency trees capture the grammatical relations between words in a sentence and are produced using a dependency parser and a dependency language. In a dependency tree, each word in a sentence corresponds to a node in the tree and is in one or more syntactic relations between the word or node exactly one other word or node. A dependency tree is a triple *T* = 〈*N*, *E*, *R* 〉, where

- *N* is the set of nodes in *T* where each node *n* ∊ *N* is a tuple containing one or more string attributes describing a word in the sentence *T* was built from, such as *word*, *lemma*, or *POS* (part of speech)
- *E* is the set of edges in *T* where each edge *e* ∊ *E* is a triple *e* = 〈*n*_*g*_, *r*, *n*_*d*_〉, where
  - *n*_*g*_ ∊ *N* is the governor or parent in relation *r*
  - *r* is a syntactic relation between the words represented by *n*_*g*_ and *n*_*d*_
  - *n*_*d*_ ∊ *N* is the dependent or child in relation *r*
- *R* ∊ *N* is the root node of *T*

**Figure 2:**
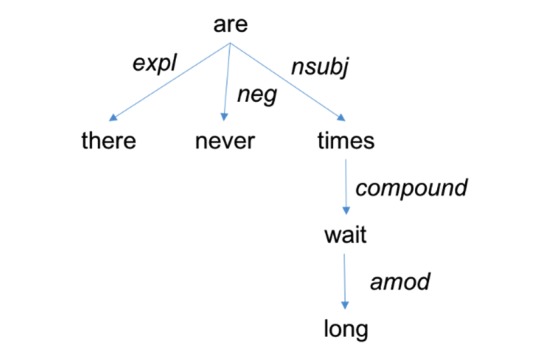
A dependency tree for the sentence "There are never long wait times".

Figure 2 shows a sample dependency tree for the sentence “there are never long wait times.” The string representation of this tree, including the parts of speech for its words, is as follows:

[are/VBP expl>there/EX neg>never/RB nsubj>[times/NNS compound>[wait/NN amod>long/JJ]]]

To match patterns against dependency trees, we used Stanford semgrex utility [32]. In the following, we explain some of the basics of semgrex patterns that help the reader understand patterns presented in this study using descriptions and examples from the Chambers et al study [32]. Semgrex patterns are composed of nodes and relations between them. Nodes are represented as *{attr1:value1;attr2:value2;…}* where attributes (attr) are regular strings such as *word*, *lemma,* and *pos,* and values can be strings or regular expressions marked by “/”s. For example, {lemma:run;pos:/VB.*/} means any verb form of the word *run*. Similar to “.” in regular expressions, *{}* means any node in the graph. Relations in a semgrex have 2 parts: the relation symbol, which can be either *<* or *>* and optionally the relation type (ie, *nsubj* and *dobj*). In general, *A<reln B* means *A* is the dependent of a relation (*reln)* with *B*, whereas *A>reln B* means *A* is the governor of a *reln* with *B*. Indirect relations can be specified by the symbols *>>* and *<<*. For example, *A<<reln B* means there is some node in a dep->gov chain from *A* that is the dependent of a *reln* with *B*. Relations can be strung together with or without using the symbol *&*. All relations are relative to first node in string. For example, *A>nsubj B>dobj D* means *A* is a node that is the governor of both an *nsubj* relation with *B* and a *dobj* relation with *D*. Nodes can be grouped with parentheses. For example, *A>nsubj (B>dobj D)* means *A* is the governor of an *nsubj* relation with *B*, whereas *B* is the governor of a *dobj* relation with *D*. A sample pattern that matches the tree in Figure 2 can be:

{} >neg {} >> ({word:wait} > {word:long})

Using the Stanford CoreNLP Java library [33], our proposed classifier builds a dependency tree from a given sentence and determines whether any of a list of semgrex patterns matches any part of the tree.

### Proposed Dependency Tree–Based Classifier

Our DTC algorithm is trained on a labeled dataset of sentences as described in the Problem Definition section. On a high level, given a sentence in training dataset *T*, the classifier generates a dependency tree using the Stanford Neural Network Dependency Parser [34] and extracts semgrex patterns from the dependency tree. These patterns are assigned the same class as the training sentence. When classifying a new sentence, the classifier generates the sentence’s dependency tree and assigns a class label to the sentence based on which patterns from the training set match the dependency tree.

In more detail, the classifier’s training algorithm generates a sorted list of semgrex patterns, each with an associated class label, from a training dataset *T* and integer parameters *n*_*i*_^*x*^, *n*_*i*_^*y*^, and *m*. Parameters *n*_*i*_^*x*^ and *n*_*i*_^*y*^ are the maximum number of terms (words or phrases) that will be used to generate patterns of classes *c*_*i*_^*x*^ and *c*_*i*_^*y*^, respectively. In this study, we only use words, as dependency trees capture relations between words rather than phrases.

The pattern extraction algorithm described in the Pattern Extraction section below receives as input 2 sets *W*^*x*^ and *W*^*y*^ of high-information gain words, for the “Yes” (*c*_*i*_^*x*^) and “No” (*c*_*i*_^*y*^) class labels, respectively, from where we pick nodes for the generated patterns. The intuition is that high-information gain words are more likely to allow a pattern to differentiate between the class labels. Considering all words would be computationally too expensive, and it does not offer any significant advantage as we have seen in our experiments. The information gain for *W*^*x*^ is determined by a logical copy of training dataset *T* in which class labels other than *c*_*i*_^*x*^ are given a new class label *c*_*i*_^*x*^*'*, as the words in *W*^*x*^ will be used to identify sentences of class *c*_*i*_^*x*^. This process is repeated for *W*^*y*^. Parameter *m* is the maximum number of these selected words that can be in a single pattern.

The final list of (semgrex pattern *p* and class label *c'*) pairs is sorted by the weighted accuracy of the pair on the training data, which we define below.

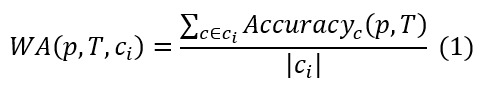



We define *Accuracy*_*c*_(*p*, *T*) as the ratio of training instances in *T* with class label *c* that were correctly handled by pattern *p*. Pattern *p*, which was paired with class label *'*, is correct if it matches an instance with class label *c'* or it does not match an instance without class label *c'*, but it is incorrect if it matches an instance without class label *c'* or it does not match an instance with class label *c'*. |*c*_*i*_| is the number of class labels in class *c*_*i*_, which is 3 for all of the classes in this study. Intuitively, weighted accuracy treats all class labels with equal importance regardless of their frequency, so patterns that perform well on sentences of often low-frequency class labels *c*_*i*_^*x*^ and *c*_*i*_^*y*^ are assigned higher rank than they would otherwise. The training algorithm is shown in Textbox 1.

Given a to-be-classified sentence, we compute its dependency tree *t* and find the highest ranked (pattern *p* and class label *c*) pair where *p* matches *t*. Then the sentence is classified as *c*. If no pattern matches the sentence, we provide 2 possibilities: the sentence can be classified as the most common class label in *T* or it can be classified by a *backup* classifier trained on *T*.

#### Parameters Setting

In all experiments, we use *n*_*i*_^*x*^=*n*_*i*_^*y*^=30, as intuitively it is unlikely that there are more than 30 words for a class that can participate in a discriminative semgrex pattern. We set *m* to 4 for all experiments, because for *m*>4, it becomes too computationally expensive to compute all patterns.

#### Pattern Extraction in the Dependency Tree Classifier Algorithm

##### Overview

Given a dependency tree, we now describe how to extract patterns. Note that we repeat the pattern extraction for the “Yes” and “No” class labels, using *W*^*x*^ and *W*^*y*^, respectively (*W* in this section refers to *W*^*x*^ or *W*^*y*^). We extract semgrex patterns from a dependency tree *t* with class label *c* using a set of high-information gain words *W* and a maximum number of words *m*. The algorithm returns a set of patterns extracted from *t* made from up to *m* words in *W*.

The rationale for only working with high-information gain words is that we want to generate high-information gain patterns. We also want to preserve negations as they have a great impact to the accuracy of the patterns. If a low information gain word is negated, we replace it by a wildcard (*), which we found to be a good balance for these 2 goals. Each pattern *p* is associated with *c* such that a new sentence that matches *p* is classified as *c*. Textbox 2 describes the pattern extraction algorithm.

**The dependency tree classifier’s training algorithm.**

1. train(*T*, *n*_*i*_^*x*^, *n*_*i*_^*y*^, *m*):
2.    *P*=list of semgrex patterns used for classification, initially empty
3.    for each class label *c* in {*c*_*i*_^*x*^, *c*_*i*_^*y*^}:
4.       *D*=set of dependency trees for sentences in *T* with class label *c*
5.       *T*_*c*_=copy of *T* with all non-*c* class labels given a new class label *c'*
6.       *W*=set of top *n*_*c*_ words *w* in *T*_*c*_ by information gain
7.       for each tree *t* in *D*:
8.          add all semgrex patterns from extract(*W*, *t*, *c*, *m*) to *P*
9.    test each pattern in *P* on *T*
10.    sort *P* by the weighted accuracy of each semgrex pattern tested on *T* in descending order
11.    return *P*

**Pattern extraction algorithm.**

1. extract(*W*, *t*, *c*, *m*):
2.    *P*=set of patterns, initially empty
3.    *S*=stack of (tree, word set) pairs, initially empty
4.    for each combination *C* of words in *W* with |*C* |==min(|*W*|, *m*)
5.       *S*.push((*t*, *C*))
6.    while *S* is not empty:
7.       (*t'*, *C*)=S.pop()
8.       *t'*=prune(*t'*, *C*)
9.       *n*=root of *t'*
10.       while *n*==* and *n* has exactly 1 child:
11.          *n*=child of *n*
12.       *t'*=subtree of *t'* with root *n*
13.       remove each “*” node *n'* in *t'* with exactly 1 child *c'*, and make the parent of *n'* the parent of *c'* with an *indirect* relation
14.       add (pattern(*t'*), *c*) to *P*
15.       for each combination *C'* of non-* words in *t'* with |*C'*| > 1:
16.          *S*.push((*t'*, *C'*))
17.    return *P*

1. prune(*t*, *W*):
2.    *t'*=copy of *t*
3.    recursively prune from *t'* leaves that do not start with any word in *W* and are not in a negation relation
4.    for each node *n* in *t'*:
5.       if *n* does not start with any word in *W*:
6.          *n*=*
7.    return *t'*

##### Details

The algorithm first creates a copy *t'* of *t* for each combination *C* of *m* words in *W* and pushes each (*t'*, *C*) pair onto a stack. For each (*t'*, *C*) popped from the stack, we execute the following steps:

1. Create initial subtree: Prune *t'* to keep only words in *C*, negations, and intermediate “*” nodes connecting them.
2. Remove unimportant nodes: Eliminate “*” nodes from *t'* starting with the root if it is a “*” node and has exactly 1 child (the child becomes the new root of *t'* and this repeats until the root no longer meets these criteria). Subsequently, remove each “*” node *n'* in *t'* with exactly 1 child and add an indirect relation edge from the parent of *n'* to the child of *n'.*
3. Add subpatterns: If (*pattern*(*t'*), *c*) is not already in *P*, add (*pattern*(*t'*), *c*) to the set of patterns *P*, and then push(*t'*, *c'*) onto the stack for each combination *C'* of 2 or more non-* words in *t'*.

The algorithm then moves on to the next item on the stack. Once the stack is empty, we return the resulting set of patterns and their associated class labels.

The *prune*(*t*, *w*) procedure recursively removes leaf nodes that do not start with any word in *W* and are not in a negation relation with their parents. Intermediate nodes that connect the remaining nodes and do not start with any word in *W* are replaced by *. The pattern(*t*) procedure converts a dependency tree *t* to its semgrex format representation. Each “*” node is represented by an empty node *{}*, and most relations are represented by the generic *>* or *>>* relations (for direct and indirect relations, respectively), which match any type of relation. An exception to this is the negation relation, which is preserved in the semgrex pattern as the *>neg* token.

##### Example

**Figure 3:**
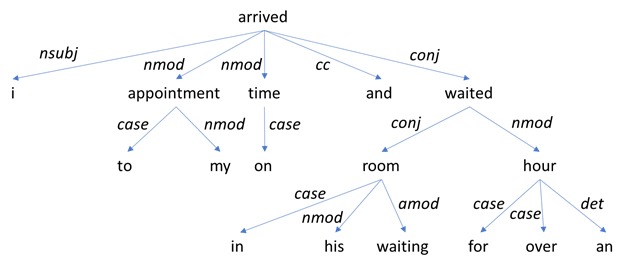
Dependency tree for the sentence "I arrived to my appointment on time and waited in his waiting room for over an hour".

Consider a sentence from the doctor review dataset class *c*_*8*_ (wait time), “I arrived to my appointment on time and waited in his waiting room for over an hour,” which has class label *c*_*8*_^*y*^ (long wait). The dependency tree generated from this sentence is shown in Figure 3.

Among the patterns extracted from this tree are:

1. {} > {word:/time.*/} >> {word:/hour.*/}
2. {word:/arrived.*/} > {word:/time.*/}
3. {} > {word:/time.*/} > ({} > {word:/room.*/} > {word:/hour.*/})
4. {word:/arrived.*/} >> {word:/hour.*/}

Pattern 1 means that some node has a direct descendant *time* and an indirect descendant *hour*. Pattern 2 means that *time* is a direct descendant of *arrived*. Pattern 3 means that some node has 2 direct descendants; 1 is *time* and the other is some other node that has direct descendants *room* and *hour*. Finally, pattern 4 means that *hour* is an indirect descendant of *arrived*.

## Results

### Classifiers Employed

We consider 3 types of classifiers:

1. *Statistical bag-of-words classifiers*, which view the documents as bags of keywords:
  - Random Forests (*RF)*: RF, as implemented in Scikit-learn by Pedregosa et al [35]. Documents are represented with TF-IDF using n grams of 1 to 3 words, a minimum document frequency of 3%, up to 1000 features, stemming, and omission of stop words. The classifier uses 2000 trees. All other parameters are given their default values from [35].
  - *SVM*: C-support vector classifier as implemented in Scikit-learn by Pedregosa et al [35], which is based on the implementation from the study by Chang and Lin [36]. Documents are represented with TF-IDF using the same parameters as with random forest. The parameters for the classifier are given their default values from Scikit-learn by Pedregosa et al [35].
2. *Deep learning classifiers:*
  - *CNN* or *CNN-W* (CNN with Word2Vec): We use 2 variants of the CNN implementation by Britz [37]. Both use the default parameters. The first variant is initialized with a random uniform distribution, as in the CNN implementation by Britz [37]. The second is initialized with values from the Word2Vec model implementation from Gensim by Rehurek and Sojka [38].
  - *D2V-NN* (Doc2Vec Nearest Neighbor): A nearest neighbor classifier that uses the Doc2Vec model [39] implementation from Gensim by Rehurek and Sojka [38]. Documents are converted to paragraph vectors and classified according to the nearest neighbor using cosine similarity as the distance function.
  For CNN-W and D2V-NN, the Word2Vec and Doc2Vec models, respectively, are trained on an unlabeled set of 8,977,322 sentences from the collected doctor reviews that were not used to create the labeled dataset.
3. *NLP classifiers*, which exploit the dependency trees of a review’s sentences:
  - *Matsumoto*: We implemented the method described in the study by Matsumoto et al [21] using the best-performing combination of features from their experiment using the Internet Movie Database dataset from the study of Pang and Lee [40], that is, unigrams, bigrams, frequent subsequences, and lemmatized frequent subtrees. For POS tagging before the step in frequent subsequence generation that splits sentences into clauses, our implementation uses the Stanford parser [41]. We use the dependency parser by Chen and Manning [34] to generate dependency trees for frequent subtree generation. For the SVM, we use the implementation from Pedregosa et al’s Scikit-learn with a linear kernel and all other parameters given their default values from [35]. All parameters related to frequent subsequence and subtree generation are the same as described in the study by Matsumoto et al [21].
  - *DTC*: As described in the Methods section.

### Variants of Dependency Tree Classifier

We consider the following variants of our DTC text classifier:

*DTC*: as described above, with sentences not matching any pattern classified as the most common class label in the training data.

*DTC*_*RF*_: Sentences not matching any pattern are classified by a random forests classifier trained on the training data for each class.

*DTC*_*CNN-W*_: Sentences not matching any pattern are classified by a CNN-W text classifier (as defined above) trained on the training data for each class.

### Experiments

We performed experiments with the classifiers on each class of the doctor review dataset using 10-fold cross validation. To evaluate their performance, we use weighted accuracy. For a trained classifier *C* and dataset *D* of class *c*_*i*_, we define this as shown below.

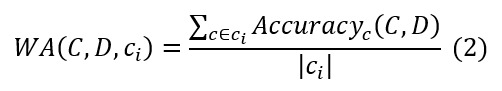



*Accuracy*_*c*_(*C*, *D*) is the ratio of sentences in *D* with class label *c* that were classified correctly by *C*. As before, |*c*_*i*_| is 3, the number of class labels in class *c*_*i*_. We use weighted accuracy in our experiments as it places more importance on less frequent class labels, whereas regular accuracy is often above 90% because of the high number of instances labeled *c*_*i*_^*0*^ for each *c*_*i*_.

The results of our experiments are shown below. In Table 3, we see that DTC_CNN-W_ has better weighted accuracy than at least 4 baselines in each class. On average, it performs 2.19% better than the second-best method, the Matsumoto classifier ([57.05%-55.83%]/55.83%=2.19%). We also observe that both the deep learning classifiers (CNN, CNN-W, and D2V-NN) and NLP classifiers (Matsumoto and DTC variants) tend to perform better than the bag-of-words classifiers (RF and SVM). This is expected as the deep learning and NLP classifiers take advantage of information in sentences such as word order and syntactic structure that cannot be expressed by a bag-of-words vector.

Next, we further examine the performance of the top 3 classifiers, CNN-W, Matsumoto, and DTC_CNN-W_. Table 4 shows the ratio of review sentences with class label *c*_*i*_^*x*^ or *c*_*i*_^*y*^ that were classified correctly in our experiments. Note that this is the *Accuracy*_*c*_(*C*, *D*) measure described above. DTC_CNN-W_ generally outperforms the other classifiers with this measure; notable exceptions are *c*_*6*_^*y*^ (bad staff), *c*_*7*_^*x*^ (long visit time), and *c*_*8*_^*y*^ (long wait time), where substantial numbers of sentences with these class labels were misclassified with the opposite label: 26.98% of *c*_*6*_^*y*^ sentences were misclassified as *c*_*6*_^*x*^ (good staff), 38.03% of *c*_*7*_^*x*^ sentences were misclassified as *c*_*7*_^*y*^ (short visit time), and 43.22% of *c*_*8*_^*y*^ sentences were misclassified as *c*_*8*_^*x*^ (short wait time). Finally, Table 5 shows the ratio of review sentences classified as *c*_*i*_^*x*^ or *c*_*i*_^*y*^ (ie, a classifier predicted their class labels as *c*_*i*_^*x*^ or *c*_*i*_^*y*^) that were classified correctly. By this measure, DTC_CNN-W_ performs poorly compared with CNN-W and Matsumoto. Although the DTC algorithm’s semgrex patterns classify more sentences as *c*_*i*_^*x*^ or *c*_*i*_^*y*^, many of these classifications are incorrect. In the next section, we discuss reasons for some of these misclassifications.

**Table 3:** Weighted accuracy of classifiers on doctor review dataset.

Classifier	*c_1_* (%)	*c_2_* (%)	*c_3_* (%)	*c_4_* (%)	*c_5_* (%)	*c_6_* (%)	*c_7_* (%)	*c_8_* (%)	Average (%)
CNN^a^	42.06	56.69	42.75	51.45	47.81	61.42	55.38	60.93	52.31
CNN-W^b^	49.89	*59.68^c^*	44.30	53.53	49.71	64.04	54.29	63.51	54.87
D2V-NN^d^	38.83	45.16	38.00	42.25	41.44	42.19	41.04	43.64	41.57
Matsumoto	45.76	59.63	45.89	53.40	49.89	*66.45*	57.24	*68.36*	55.83
RF^e^	40.78	42.00	34.76	37.29	41.62	52.88	45.65	51.66	43.33
SVM^f^	33.33	35.77	33.33	33.33	33.33	48.94	33.33	48.07	37.43
DTC^g^	51.72	50.48	41.27	47.23	38.49	54.31	*60.90*	65.91	51.29
DTC_RF_	*54.00*	46.64	39.19	47.29	40.20	56.15	60.57	58.05	50.26
DTC_CNN-W_	53.89	59.37	*48.66*	*57.98*	*50.77*	61.43	56.63	67.67	*57.05*

^a^CNN: Convolutional Neural Network.

^b^CNN-W: Convolutional Neural Network with Word2Vec.

^c^The highest value for each *c*_*i*_ is italicized for emphasis.

^d^D2V-NN: Doc2Vec Nearest Neighbor.

^e^RF: Random Forests.

^f^SVM: Support Vector Machine.

^g^DTC: dependency tree classifier.

**Table 4:** Per-label accuracy of top 3 classifiers on doctor review dataset for each *c*_*i*_^*x*^ and *c*_*i*_^*y*^.

Label and classifier		*c_1_* (%)	*c_2_* (%)	*c_3_* (%)	*c_4_* (%)	*c_5_* (%)	*c_6_* (%)	*c_7_* (%)	*c_8_* (%)
***c_1_^x^***									
	CNN-W^a^	31.37%	57.22%	0.00%	47.62%	40.54%	60.69%	45.07%	40.85%
	Matsumoto	13.73%	57.04%	*4.00%* ^b^	48.57%	41.16%	59.16%	*52.11%*	47.89%
	DTC^c^_CNN-W_	*33.33%*	*59.69%*	*4.00%*	*51.11%*	*48.02%*	*64.89%*	39.44%	*71.83%*
***c_1_^y^***									
	CNN-W	19.05%	27.35%	34.48%	15.44%	13.36%	35.42%	18.99%	50.75%
	Matsumoto	23.81%	27.65%	35.00%	13.24%	12.93%	*43.32%*	20.25%	*57.79%*
	DTC_CNN-W_	*33.33%*	*48.24%*	*47.51%*	*38.97%*	*25.00%*	27.52%	*35.44%*	35.68%

^a^CNN-W: Convolutional Neural Network with Word2Vec.

^b^For each *c*_*i*_, the highest value for both *c*_*i*_^*x*^ and *c*_*i*_^*y*^ are italicized for emphasis.

^c^DTC: dependency tree classifier.

**Table 5:** Ratio of sentences classified by the top 3 classifiers as *c*_*i*_^*x*^ or *c*_*i*_^*y*^ that were classified correctly.

Label and classifier		*c_1_* (%)	*c_2_* (%)	*c_3_* (%)	*c_4_* (%)	*c_5_* (%)	*c_6_* (%)	*c_7_* (%)	*c_8_* (%)
***c_1_^x^***									
	CNN-W^a^	34.78%	*60.19%* ^b^	0.00%	62.50%	50.26%	66.81%	57.14%	65.91%
	Matsumoto	*46.67%*	43.40%	*50.00%*	*66.23%*	*55.31%*	*71.10%*	*67.27%*	*77.27%*
	DTC^c^_CNN-W_	16.04%	41.66%	10.00%	20.69%	22.58%	43.59%	23.73%	21.52%
***c_1_^y^***									
	CNN-W	40.00%	*41.52%*	50.56%	22.83%	*28.70%*	41.27%	29.41%	59.06%
	Matsumoto	*58.82%*	34.18%	*56.52%*	*34.62%*	25.64%	*49.53%*	*53.33%*	*70.99%*
	DTC_CNN-W_	10.98%	13.50%	28.57%	13.38%	14.25%	22.90%	14.29%	29.96%

^a^CNN-W: Convolutional Neural Network with Word2Vec.

^b^For each *c*_*i*_, the highest value for both *c*_*i*_^*x*^ and *c*_*i*_^*y*^ are italicized for emphasis.

^c^DTC: dependency tree classifier.

## Discussion

### Anecdotal Examples

In this section, we show some specific patterns generated by our algorithm along with some actual review sentences that match these patterns. The semgrex pattern {} >neg {} >> ({word:/wait.*/} > {word:/long.*/}) was generated from a sentence with class label *c*_*8*_^*x*^ (short wait) in class *c*_*8*_ (wait time) in the doctor review dataset. It consists of a node that has 2 descendants: another generic node in a direct negation relation and *wait* in an indirect relation. The word *wait* has 1 direct descendant, the word *long*. The following is an example of a correctly matched sentence: “You are known by name and never have to wait long.” This is an incorrectly matched one: “As a patient, I was not permitted to complain to the doctor about the long wait, placed on hold and never coming back to answer call.” We see that it contains the words *long* and *wait*, as well as a negation (the word *never*); however, the negation is not semantically related to the *long wait* the author mentioned. Providing additional training data to the classifier may prevent such misclassifications by finding a pattern (or improving the rank of an existing pattern) that more appropriately makes such distinctions.

### Limitations

In addition to the incorrect handling of negation described above, another limitation of our algorithm is that some sentences of a particular class can be sufficiently similar to sentences from another class, which may lead to misclassifications. Some examples of this can be seen in class *c*_*6*_ (staff). Specifically, some sentences referring to a doctor (rather than staff members) were incorrectly classified as *c*_*6*_^*x*^ (good staff) or *c*_*6*_^*y*^ (bad staff). For example, “Dr. Fang provides the very best medical care available anywhere in the profession” and “Dr. Overlock treated me with the utmost respect,” which clearly refer to doctors rather than staff and should have been classified as *c*_*6*_^*0*^ (no mention of staff). The DTC algorithm generated some patterns for *c*_*6*_^*x*^ that focus on positive statements for a person but miss the requirement that this person is staff. In the case of the above sentences, they were matched by {} >> {word:/dr.*/} >> {word:/best.*/} and {} >> {word:/with.*/} >> {word:/dr.*/}, respectively, which both erroneously include the word *dr*. More work is needed to address such tricky issues.

### Conclusions

In this paper, we study the doctor reviews classification problem. We evaluate several existing classifiers and 1 new classifier. A key challenge of the problem is that features may be complex entities, for which polarity is not necessarily compatible with traditional positive or negative sentiment. Our proposed classifier, DTC, uses dependency trees generated from review sentences and automatically generates patterns that are then used to classify new reviews. In our experiments on a real-world doctor review dataset, we found that DTC outperforms other text classification methods. Future work may build upon the DTC classifier by also incorporating other NLP structures, such as discourse trees [42], to better capture the semantics of the reviews.

## Abbreviations

Attr: attribute
CNN: Convolutional Neural Network
CNN-W: Convolutional Neural Network initialized with values from a Word2Vec model
D2V-NN: nearest neighbor classifier that uses the Doc2Vec model
DTC: dependency tree classifier
NLP: natural language processing
POS: part of speech
RF: random forests
S: sentiment
SVM: Support Vector Machine
TF-IDF: term frequency-inverse document frequency
T: target
